# Injection-site pain after switching from somatrogon to somapacitan in children reporting pain or discomfort during once-weekly growth hormone therapy: a prospective single-arm switch study

**DOI:** 10.3389/fendo.2026.1849487

**Published:** 2026-08-31

**Authors:** Hideyuki Iwayama, Kohei Kawahara, Sachiko Kitagawa, Ryosuke Miyamoto, Junko Takagi, Yoshinori Ito, Akihisa Okumura

**Affiliations:** 1 Department of Pediatrics, Aichi Medical University, Nagakute, Aichi, Japan; 2 Hoshigaoka Growth Clinic, Nagoya, Aichi, Japan; 3 Department of Pediatrics, Daiyukai General Hospital, Ichinomiya, Aichi, Japan; 4 Division of Endocrinology and Metabolism, Department of Internal Medicine, Aichi Medical University, Nagakute, Aichi, Japan

**Keywords:** injection-site pain, once-weekly growth hormone, self-reported pain, shared decision-making, somapacitan, somatrogon, switch study, tolerability

## Abstract

**Background:**

Once-weekly growth hormone (GH) formulations, such as somatrogon and somapacitan, may reduce the burden of treatment, but injection-site pain can affect treatment preference. Clinical data on patient-reported injection-site pain and preference after switching between once-weekly GH formulations remain limited, particularly in children who experience injection-site pain.

**Objective:**

To describe changes in perceived injection-site pain and treatment preference after switching from somatrogon to somapacitan in children who reported injection-site pain or discomfort during somatrogon therapy.

**Methods:**

This prospective single-arm switch study included 19 children with GH deficiency who had received somatrogon for at least 4 weeks, reported injection-site pain or discomfort during routine clinical care, and agreed to switch to somapacitan. Pain scores for prior somatrogon use and current somapacitan use were assessed once after at least 4 weeks of somapacitan treatment, using the Numerical Rating Scale and/or Faces Rating Scale according to age. A 5-point Likert questionnaire evaluated device usability, pain at needle insertion, pain during injection, pain after injection, and treatment preference. Paired t-tests and binomial tests were used for the main analyses, and Wilcoxon signed-rank tests were performed as sensitivity analyses for paired pain-score comparisons.

**Results:**

The mean age was 9.6 ± 2.6 years. Diagnoses included isolated GH deficiency (n = 17) and panhypopituitarism after craniopharyngioma surgery (n = 2). Pain scores decreased after switching (Numerical Rating Scale: 6.5 ± 2.6 vs 1.6 ± 1.7; Faces Rating Scale: 3.8 ± 1.1 vs 1.1 ± 1.1; both p < 0.001). All participants preferred continued use of somapacitan (p < 0.001), and questionnaire responses favored somapacitan for pain at needle insertion and pain during injection.

**Conclusion:**

In children who reported injection-site pain or discomfort during somatrogon therapy and agreed to switch, pain scores and treatment preference improved after switching to somapacitan. These findings support considering a switch to another once-weekly GH formulation when injection-site pain affects treatment preference.

## 1 Introduction

Successful growth hormone (GH) therapy in children needs good adherence to long-term subcutaneous injections. Several factors may interfere with adherence, including injection frequency and injection-site pain. However, injection-site pain — a key factor influencing adherence — has received limited attention in pediatric endocrinology. Most clinical studies of GH therapies have prioritized growth outcomes, and few have systematically evaluated pain differences between products. This gap suggests a broader tendency to underestimate the subjective experience of treatment-related pain in children. Addressing this gap may improve patient satisfaction and adherence to long-term GH therapy. Therefore, greater attention to injection-site pain is needed to support better adherence and more patient-centered care.

Once-weekly GH products such as somatrogon and somapacitan reduce the burden of daily injections. Although both drugs have demonstrated non-inferiority to daily GH in growth outcomes (1–3), somatrogon has been reported to be associated with more injection-site pain than daily GH (3, 4). Differences in formulation- and injection-related factors, including pH, osmolality, buffer composition, viscosity, injection volume, and needle gauge, may influence injection-site pain (5, 6).

While once-weekly GH therapies are increasingly used in clinical practice, few studies have examined patient-reported injection-site pain and product preference after switching between once-weekly GH formulations in real-world settings. Generating such data may support shared decision-making between clinicians, patients, and caregivers.

This study aimed to describe changes in perceived injection-site pain and treatment preference after switching from somatrogon to somapacitan in children who reported injection-site pain or discomfort during somatrogon therapy.

## 2 Materials and methods

### 2.1 Study design

This was a prospective, single-center, single-arm switch study conducted at Aichi Medical University Hospital, approved by the ethics committee of Aichi Medical University (approval number: 24-794).

### 2.2 Participants

Eligible patients were consecutively screened during routine clinical care. At the start of the study, 21 children with GH deficiency aged ≥3 years had received somatrogon for at least 4 weeks at our hospital. Some patients had previously received daily GH therapy before switching to somatrogon, whereas others initiated GH treatment with somatrogon. In this study, GHD was defined as height below −2 SD together with subnormal peak GH responses in two pharmacological GH stimulation tests. The cutoff values were peak GH ≤6 ng/mL for the L-dopa, clonidine, or arginine stimulation tests and peak GH ≤16 ng/mL for the GHRP-2 stimulation test, according to the diagnostic criteria used in Japan. Serum IGF-1 was used as supportive clinical information but was not used as the sole diagnostic criterion. Patients with idiopathic short stature or constitutional delay of growth and puberty who did not meet these biochemical criteria were not included in the GHD population. Somatrogon was initiated according to routine clinical practice without the 6-week step-up dosing schedule used in the Japanese phase 3 trial (3). During routine clinical visits, patients and their parents or guardians were verbally asked by the physician whether the child reported injection-site pain or discomfort during somatrogon treatment. Pain intensity was not graded using a standardized pain scale at the time of screening, and neither a minimum pain score nor a predefined duration of pain persistence was required for inclusion. Eligibility was based on patient- or parent-reported injection-site pain or discomfort during somatrogon therapy in routine clinical care and agreement to switch to somapacitan.

### 2.3 Exclusion criteria

Exclusion criteria included the use of medications known to influence pain perception or GH response beyond physiologic hormone replacement. No participants were excluded on this basis. Physiologic replacement doses of levothyroxine, hydrocortisone, or desmopressin were allowed. Exclusion criteria also included a history of severe allergic or systemic adverse reactions to somatrogon, somapacitan, or similar protein-based therapies, or inability to self-report pain.

### 2.4 Explanation before enrollment

Enrollment was limited to patients who had reported injection-site pain or discomfort during somatrogon treatment in routine clinical care. The study protocol was explained to these eligible participants and their parents or guardians by the treating physician during routine clinical care. At the time of explanation, families were informed that the study aimed to evaluate injection-site pain and treatment preference after switching from somatrogon to somapacitan in children who had reported injection-site pain or discomfort during somatrogon therapy. They were also informed that participation was voluntary, that declining participation would not affect their usual medical care, and that continuing somatrogon without switching was an available option. After experiencing at least 4 weeks of somapacitan treatment, participants and families could choose either GH formulation for continued use.

No suggestive wording was used to imply that somapacitan would cause less pain than somatrogon. Families were not told that the alternative formulation was expected to be less painful; rather, they were informed that the study would evaluate their own experience after switching. Written informed consent was obtained from all parents or legal guardians.

### 2.5 Injection information

Somatrogon (Ngenla^®^, Pfizer) was administered using the 60 mg/1.2 mL prefilled pen (50 mg/mL). Each 1.2 mL contains 60 mg of somatrogon-ghla, along with citric acid monohydrate (0.3 mg), histidine (1.9 mg), metacresol (4 mg), poloxamer 188 (2 mg), sodium chloride (10 mg), and sodium citrate (2.8 mg) in water for injection, with a pH of approximately 6.6 (7). For example, a child weighing 30 kg received a somatrogon dose of 19.8 mg, corresponding to an injection volume of approximately 0.40 mL.

Somapacitan (Sogroya^®^, Novo Nordisk) was administered using the 15 mg/1.5 mL prefilled pen (10 mg/mL). Each mL contains 10 mg of somapacitan-beco, histidine (0.68 mg), mannitol (44 mg), phenol (4 mg), and poloxamer 188 (1 mg) in water for injection, with a pH of approximately 6.8 (8). For example, a child weighing 30 kg received a somapacitan dose of 4.8 mg, corresponding to an injection volume of approximately 0.48 mL.

According to the Japanese prescribing information (9, 10), the osmotic ratios of somatrogon and somapacitan are approximately 1.2 and 1.0 relative to isotonic saline, respectively. The somatrogon injection uses a 32-gauge needle, whereas the somapacitan injection uses a thinner 34-gauge needle, according to the manufacturers’ instructions.

### 2.6 Assessments

During routine clinical care, all eligible patients and their parents or guardians were asked in Japanese whether the child experienced injection-site pain or discomfort during somatrogon treatment. The same wording was used for all patients as far as possible. Children aged 6 years or older completed the pain scales themselves, whereas for children younger than 6 years, parents or guardians completed the scales based on discussion with the child about the child’s pain experience. Pain scores for prior somatrogon use and current somapacitan use were collected once after switching to somapacitan, after at least 4 weeks of somapacitan treatment. Therefore, it is important to note that pain scores for somatrogon were assessed retrospectively and relied on recall, whereas pain scores for somapacitan reflected the current treatment experience.

The minimum post-switch exposure period of 4 weeks was chosen because both somatrogon and somapacitan are administered once weekly, and this period corresponds to at least four injections. We considered this the minimum period required for patients and families to experience repeated somapacitan injections and to provide an initial assessment of injection-site pain after switching. This criterion was not intended to standardize the exposure periods between the two formulations or to evaluate long-term adaptation to somapacitan.

Height and weight measurements obtained closest to the date of switching from somatrogon to somapacitan were used to calculate BMI. BMI SDS was calculated according to age and sex using the Body Size Index Calculator version 3.3 developed by the Japanese Society for Pediatric Endocrinology and the Japanese Association for Human Auxology, based on Japanese pediatric LMS reference data (11).

#### 2.6.1 Pain scales

Faces Rating Scale (FRS, 0–5) (12) and Numeric Rating Scale (NRS, 0–10) (13) were used to assess injection-site pain. Children aged 6 years or older completed the pain scales themselves, whereas for children younger than 6 years, parents or guardians completed the scales based on discussion with the child about the child’s pain experience. Some pain-scale responses could not be obtained when the injection was administered while the child was asleep.

#### 2.6.2 Structured questionnaires

The structured questionnaire evaluated ease of use, pain at needle insertion, pain during injection, pain after injection, burden of two injections due to insufficient residual volume, and preference for continued use. For each item, participants were asked to indicate which formulation they preferred after experiencing both treatments. For pain-related items, preference indicated the formulation associated with less pain. Responses were recorded on a 5-point Likert scale: strongly prefer somatrogon, somewhat prefer somatrogon, no preference, somewhat prefer somapacitan, or strongly prefer somapacitan.

### 2.7 Statistical analysis

Paired t-tests were used to compare pain scores before and after switching, and binomial tests were used for categorical preferences. Wilcoxon signed-rank tests were additionally performed as sensitivity analyses for the paired comparisons of NRS and FRS scores, given the small sample size and ordinal nature of the pain scales. Spearman’s rank correlation coefficients were used for exploratory analyses of the associations between BMI SDS and retrospectively reported somatrogon-related NRS and FRS scores. For binomial testing, responses were grouped into somapacitan preference (somewhat or strongly prefer somapacitan) versus no somapacitan preference (strongly or somewhat prefer somatrogon, or no preference). Continuous variables were summarized using both mean ± standard deviation (SD) and median with range because of the small sample size. No formal *a priori* power calculation was performed because this was an exploratory single-center switch study. As a *post hoc* assessment, statistical power was estimated for the paired comparisons using the observed mean and SD of the paired differences, with a two-sided alpha level of 0.05.

## 3 Results

At the start of the study, 21 children had received somatrogon for at least 4 weeks at our hospital. Of these, 9 had previously received daily GH therapy before switching to somatrogon, whereas 12 had initiated GH treatment with somatrogon. During routine clinical care, 21 patients reported some degree of injection-site pain or discomfort during somatrogon treatment. Of these, 19 patients agreed to switch to somapacitan and were enrolled in the study. The remaining two patients also reported injection-site pain or discomfort but were not enrolled: one had become accustomed to the pain and did not wish to switch formulations, and the other had autistic traits and did not agree to a change in injection formulation. The 19 enrolled children included 15 males and 4 females, with a mean age of 9.6 ± 2.6 years [median, 9.2 years; range, 4.7–15.0 years]. Diagnoses included isolated GH deficiency in 17 patients and panhypopituitarism after craniopharyngioma surgery in 2 patients. Among the 19 enrolled patients, 8 had previously received daily GH therapy before switching to somatrogon, whereas 11 had initiated GH treatment with somatrogon. Among the 8 patients with prior daily GH exposure, the duration of daily GH treatment was 205.7 ± 104.5 weeks [median, 207.6 weeks; range, 56.9–382.4 weeks]. The treatment durations for somatrogon and somapacitan were 92.0 ± 76.9 weeks [median, 64.3 weeks; range, 33.9–308.6 weeks] and 9.5 ± 6.9 weeks [median, 8.0 weeks; range, 3.9–32.0 weeks], respectively. At the time of switching from somatrogon to somapacitan, the mean BMI was 17.51 ± 2.11 kg/m² [median, 17.16 kg/m²; range, 13.52–22.07 kg/m²], and the mean BMI SDS was 0.18 ± 0.78 [median, 0.18; range, −1.63–1.51]. In an exploratory analysis, BMI SDS at the time of switching was not significantly associated with retrospectively reported somatrogon-related NRS scores (Spearman’s rho = −0.109, p = 0.677) or FRS scores (Spearman’s rho = 0.300, p = 0.226).

Pain scores for both treatments, assessed after at least 4 weeks of somapacitan treatment, were significantly lower after switching to somapacitan. Complete paired data were available for 17 patients for NRS and 18 patients for FRS. Missing pain-scale responses occurred when injections were administered while the child was asleep, making pain assessment infeasible. NRS decreased from 6.5 ± 2.6 during somatrogon use to 1.6 ± 1.7 during somapacitan use (mean paired difference, 4.94; 95% CI, 3.37–6.51; p < 0.001), and FRS decreased from 3.8 ± 1.1 to 1.1 ± 1.1 (mean paired difference, 2.69; 95% CI, 1.86–3.52; p < 0.001). *Post hoc* power analysis based on complete paired observations showed that the observed changes in pain scores had high statistical power. For the NRS, the mean paired difference was 4.94 with an SD of 3.05 among 17 patients with complete paired data, corresponding to an effect size of 1.62 and a *post hoc* power of >99.9%. For the FRS, the mean paired difference was 2.69 with an SD of 1.67 among 18 patients with complete paired data, corresponding to an effect size of 1.61 and a *post hoc* power of >99.9%. Sensitivity analyses using Wilcoxon signed-rank tests showed results consistent with the paired t-tests, with significant reductions in both NRS and FRS scores (both p < 0.001).

To explore whether pain scores were lower in patients with longer prior somatrogon exposure, we examined the association between somatrogon treatment duration and retrospectively reported somatrogon-related pain scores. Somatrogon treatment duration was not significantly associated with NRS scores for somatrogon (Spearman’s rho = 0.206, p = 0.429) or FRS scores for somatrogon (Spearman’s rho = −0.043, p = 0.866).

Structured questionnaires (**Table 1**) showed that somapacitan was associated with significantly less pain at needle insertion (p < 0.001) and pain during injection (p < 0.001). In contrast, there was no significant difference in ease of use (p = 0.324), pain after injection (p = 0.084), or the burden associated with administering two injections due to insufficient residual volume (p = 0.968). Regarding overall preference, all participants selected somapacitan to continue after the study period (p < 0.001, exact binomial test).

**Table 1:** Structured questionnaire responses after experiencing both somatrogon and somapacitan.

Questionnaire item	Strongly prefer somatrogon	Somewhat prefer somatrogon	No preference	Somewhat prefer somapacitan	Strongly prefer somapacitan	p
Ease of use	0	1	7	4	7	0.324
Less pain at needle insertion	0	0	2	5	12	< 0.001
Less pain during injection	0	0	0	2	17	< 0.001
Less pain after injection	0	0	6	0	13	0.084
Lower burden associated with two injections due to insufficient residual volume	0	0	13	1	5	0.968
Preference for continued use	0	0	0	4	15	< 0.001

Values indicate the number of participants who selected each preference on the 5-point Likert scale.

## 4 Discussion

In this prospective single-arm switch study, children who had reported injection-site pain or discomfort during somatrogon therapy reported lower NRS and FRS scores after switching to somapacitan. Because pain perception varies widely among individuals and both the formulation and needle gauge differ between somatrogon and somapacitan, the rationale for the study design and the possible causes of the pain difference are discussed below.

### 4.1 Study design rationale

According to a recent systematic review by Junker et al. (14), prospective studies comparing injection-site pain between different formulations can be broadly categorized into three designs: (a) parallel-group head-to-head trials, (b) crossover trials, and (c) sequential within-subject trials. Parallel-group head-to-head trials compare different patient populations and therefore may be affected by between-group differences such as baseline characteristics or pain sensitivity. Crossover trials evaluate both formulations within the same individuals and randomize the order of administration to minimize sequence effects. Sequential within-subject trials also evaluate both formulations in the same individuals but follow a fixed administration order.

The present study corresponds to category (c), a sequential within-subject switch study, in which all participants received somatrogon followed by somapacitan. Although the order of administration was fixed, this design allowed within-patient assessment of pain before and after switching, while reducing the influence of between-patient variability in pain perception. However, because the study lacked randomization, counterbalancing, and comparable exposure periods, it should not be interpreted as a balanced head-to-head comparison of the two formulations.

Direct comparisons of injection-site pain between GH products are rare, even among daily GH therapies. A multicenter phase 3 randomized controlled trial reported that weekly somatrogon was associated with a higher frequency of injection-site pain compared with daily somatropin (39.4% vs 25.2%) in GH-naïve patients (1). Based on the classification by Junker (14), that study corresponds to category (a), a parallel-group head-to-head trial, which may be influenced by between-group differences such as baseline pain sensitivity. In contrast, the present study used a sequential within-subject switch design, which reduced between-patient variability but introduced limitations related to the fixed treatment sequence and unequal exposure durations.

### 4.2 Possible mechanisms underlying pain reduction

Although the study was not designed to determine the precise cause of reduced pain, several formulation- and device-related factors may have contributed to the observed differences. These include differences in pH, osmolality, buffering agents (e.g., citrate), needle gauge, device-related injection dynamics, and injection volume. Because these factors changed simultaneously when patients switched from somatrogon to somapacitan, the relative contribution of each factor cannot be determined in the present study. Therefore, the mechanisms discussed below should be interpreted as hypotheses generated by the observed clinical findings, rather than as causal findings of the study.

The effect of pH on subcutaneous injection pain was investigated by Fransson et al. (15), who compared isotonic phosphate-buffered formulations with pH values ranging from 6 to 7 and phosphate concentrations from 5 to 50 mM. The least discomfort was observed with 10 mM phosphate at pH 7. Injection of buffer at pH 6 with 50 mM phosphate caused significantly more pain than 10 mM phosphate, but no significant difference was noted between pH 6 and pH 7 when the phosphate concentration was 10 mM. These results suggest that a pH difference of 1.0 may not substantially impact injection-site pain under certain buffer conditions. In the present study, the pH difference between somatrogon and somapacitan was only 0.2. Therefore, the contribution of pH to the observed difference in injection-site pain is likely minimal.

The ideal osmolality for isotonic subcutaneous injection is approximately 300 mOsm/kg (16). Intramuscular injection of 5.8% hypertonic saline (1984 mOsm/kg) has been shown to induce greater pain compared to isotonic saline (308 mOsm/kg) (17). In our study, somatrogon had a higher osmolality (1.2 relative to isotonic saline) than that in somapacitan (1.0 relative to isotonic saline); thus, although the precise impact remains uncertain, this difference in osmolality may have contributed to the injection-site pain observed between the two formulations.

Another important consideration is the possible change in injection-site pain over time. In a Japanese phase 3 study of somatrogon, most events of injection-site pain were reported during the first 6 months of treatment (3), suggesting that the timing of assessment and treatment adaptation may influence the observed tolerability of somatrogon. In the present study, serial prospective NRS and FRS assessments were not performed during somatrogon treatment; therefore, we cannot directly evaluate the time course of pain within individual patients. However, in an exploratory analysis, longer somatrogon exposure was not significantly associated with lower retrospectively reported somatrogon-related pain scores. Thus, although adaptation over time remains possible and should be assessed prospectively in future studies, we did not observe clear evidence that longer prior somatrogon exposure was associated with lower recalled injection-site pain in this cohort. In addition, unlike the Japanese phase 3 trial, which used a 6-week step-up dosing schedule for somatrogon, the present cohort did not use a protocol-defined step-up schedule. This difference in dosing approach may have influenced tolerability during the early treatment period and should be considered when comparing the present findings with previous clinical trial data.

The frequency of reported injection-site pain or discomfort in the present source population should also be interpreted in the context of previous studies. In the Japanese phase 3 study, injection-site pain with a pain score ≥4 was reported in 16 of 22 patients receiving somatrogon (72.7%), and any injection-site pain was reported in all patients receiving somatrogon (3). A recent real-world study by Atas et al. reported injection-site pain in 13.6% of 121 Turkish children with GHD who were switched to somatrogon after at least 1 year of daily GH treatment (18). This frequency was substantially lower than that observed in the present source population. However, the treatment backgrounds differed between the studies: all participants in the study by Atas et al. had prior daily GH exposure, whereas only 9 of the 21 patients in our source population had previously received daily GH, and the remaining 12 initiated GH treatment with somatrogon. Differences in study design, symptom ascertainment, treatment history, injection practices, and population characteristics preclude direct comparison. Nevertheless, the Japanese phase 3 findings suggest that a high frequency of injection-site pain may not necessarily be unusual among Japanese pediatric patients receiving somatrogon.

Citrate-buffered formulations have been associated with injection-site pain in adult patients receiving biologic agents. A systematic review by Junker et al. (14) summarized two observational studies, two crossover/sequential trials, and three head-to-head comparison trials evaluating citrate-containing versus citrate-free biologic formulations. Among these, five studies reported reduced injection-site pain with citrate-free formulations. However, the review did not conduct a formal meta-analysis, likely due to the heterogeneity in reporting methods; pain intensity was typically presented using descriptive statistics (e.g., presence or absence of mean differences or statistical significance), and standardized outcome measures across studies were lacking. In our study, somatrogon contains citric acid monohydrate (0.3 mg) and sodium citrate (2.8 mg) as buffering agents, while somapacitan does not contain citrate-based buffers; instead, histidine is used as the buffering agent, as is also present in somatrogon. However, these data were derived mainly from adult patients receiving biologic agents, including rheumatology biologics, and cannot be directly extrapolated to pediatric patients receiving subcutaneous GH therapy. Differences in patient population, underlying disease, injection frequency, formulation, device, and pain-reporting characteristics should be considered. Therefore, the possible contribution of citrate-free buffering in the present study should be interpreted as a hypothesis supported by indirect evidence rather than as a direct causal finding.

A comparative study of site-injection pain with 32- and 34-gauge insulin pen needles reported that the 34-gauge needle was associated with significantly lower pain during insulin delivery, though pain at needle insertion did not differ significantly between the two gauges (19). In our study, somapacitan utilizes a 34-gauge needle, which is thinner than the 32-gauge needle used with somatrogon, potentially reducing pain at needle insertion and pain during injection. In addition to needle gauge itself, device-related injection dynamics may also influence injection-site pain. Rapid delivery of subcutaneous fluid can cause local tissue distension and may contribute to pain during injection. In the present study, injection speed, injection force, and actual drug delivery time were not measured. Because both products were administered using manufacturer-specified prefilled pen devices, the injection process was largely device-guided rather than manually controlled as in syringe injections. Nevertheless, differences in device mechanics, needle gauge, solution viscosity, injection resistance, or delivery time may have contributed to the observed differences in pain during injection.

Higher injection volumes have been associated with increased injection-site pain, and a maximum volume of 2 mL per dose is recommended (20). In contrast, Chantelau et al. reported that injection-site pain was unrelated to injection volumes below 0.5 mL in patients with diabetes mellitus (21). However, pain perception in patients with diabetes mellitus, in whom peripheral sensory function may differ from that of children without diabetes, may not be directly comparable to that of the pediatric GH population studied here. In the present study, injection volumes were comparable between products. For example, for a 30 kg child, the estimated injection volume was approximately 0.40 mL for somatrogon and 0.48 mL for somapacitan, and both were below 0.5 mL. Although the precise impact of injection volume remains unclear, this modest difference is unlikely to account for the observed differences in pain.

### 4.3 Clinical implications for shared decision-making

Shared decision-making between the practitioner, the patient, and their family is increasingly recommended to guide the selection of long-acting GH therapies, including the choice of formulation and delivery device, as emphasized in recent international consensus statements (4). However, its implementation may be limited in the context of GH therapy due to the lack of comparative tolerability data, physicians’ limited direct experience with the injection process, and the inherent difficulty children encounter in expressing pain verbally. These findings may help clinicians discuss switching options with patients and families when injection-site pain affects treatment acceptability.

Regardless of the specific GH formulation used, clinicians should consider providing patients and families with practical instruction on injection technique and pain-minimization strategies, including rotation of injection sites, appropriate needle handling, and, when feasible, non-pharmacological approaches such as brief cold application, vibration, or distraction. When injection-site pain remains clinically relevant despite such measures, switching to another available long-acting GH formulation may be discussed as part of shared decision-making.

This study did not systematically evaluate treatment adherence, missed injections, or growth outcomes in relation to injection-site pain. Future studies should prospectively evaluate pain, adherence, missed doses, and growth outcomes using standardized measures.

### 4.4 Limitations of this study

This study has several limitations. First, the sample size was small and the scope of comparison was limited. Although the *post hoc* power analysis indicated high power for the observed within-subject differences in pain scores, this finding should be interpreted in the context of the large observed effect size and does not overcome the limitations of the small sample size. The small cohort still limits the precision of the estimates and the generalizability of the findings. In addition, the study did not include direct comparisons with GH products other than somatrogon. Therefore, the findings cannot be generalized to switching from other daily or long-acting GH formulations to somapacitan, or to comparisons among other GH preparations.

Second, the generalizability of the findings is limited by the selected study population and clinical setting. The study enrolled only children who reported injection-site pain or discomfort during somatrogon therapy and agreed to switch to somapacitan. Therefore, the cohort was not representative of all pediatric somatrogon users, and the findings should not be interpreted as evidence of the general superiority of somapacitan over somatrogon in injection-site pain. Rather, they describe the experience of a selected group of children who reported injection-site pain or discomfort during somatrogon therapy and subsequently switched to somapacitan. In addition, two eligible patients were not enrolled: one because the child had adapted to injection-related pain, and the other because the child had autistic traits and preferred to avoid changes in routine. Furthermore, this was a single-center study conducted in an all-Japanese pediatric population. Subcutaneous tissue characteristics, injection practices, and pain-reporting norms may differ across populations and clinical settings. These factors preclude estimation of the overall frequency or severity of injection-site pain among all children treated with somatrogon and indicate that the findings should be generalized with caution to broader pediatric somatrogon users, non-Japanese populations, and other healthcare settings.

Third, the fixed sequence of switching from somatrogon to somapacitan introduces the possibility of order effects. Because all participants received somatrogon before somapacitan, the study lacked randomization and counterbalancing, and therefore cannot distinguish product-related differences from sequence-related effects.

Fourth, the exposure periods and timing of pain assessment were not standardized between the two formulations. Although the minimum post-switch exposure period of 4 weeks corresponds to at least four once-weekly somapacitan injections and was considered sufficient for an initial assessment of injection-site pain, the actual duration of somatrogon treatment was substantially longer than that of somapacitan treatment. In addition, pain scores for prior somatrogon use were collected retrospectively after switching to somapacitan. This approach may have introduced recall bias, because patients and families may have forgotten, reinterpreted, or selectively remembered their previous somatrogon-related pain experience.

Nevertheless, because pain is inherently subjective and difficult to quantify without a reference experience, assessment after exposure to both formulations may have allowed patients and families to evaluate the relative severity of injection-site pain using a common internal reference. Thus, the single post-switch assessment may have facilitated within-patient comparison of perceived pain between the two formulations. However, this does not eliminate the possibility of recall bias or replace the need for prospective assessment at predefined time points. Future studies should ideally collect pain scores prospectively at the final somatrogon injection before switching and again after an identical predefined exposure period of somapacitan, preferably within a randomized crossover design with counterbalanced treatment order.

Finally, although patients and families received standard instruction regarding GH injection technique, the study did not include a standardized counseling protocol for pain-minimization strategies such as cold application, vibration, or structured distraction. Moreover, information on whether these strategies were actually used during somatrogon injections was not systematically collected. Such non-pharmacological measures have been shown to reduce pain and fear during subcutaneous insulin injections in children (22) and may therefore have influenced the reported injection-site pain in the present study. In addition, body habitus and subcutaneous tissue thickness may affect injection-site pain. Although BMI SDS was not significantly associated with retrospectively reported somatrogon-related NRS or FRS scores in this exploratory analysis, BMI SDS is only an indirect indicator of body habitus, and subcutaneous tissue thickness and injection-site adiposity were not directly assessed. These unmeasured factors should be considered when interpreting the reported injection-site pain.

## 5 Conclusion

Among children who reported injection-site pain or discomfort during somatrogon therapy and agreed to switch, pain scores and treatment preference improved after switching to somapacitan. Although the single-arm design, selected cohort, and unequal exposure durations preclude conclusions about the general superiority of one once-weekly GH formulation over another, these findings suggest that switching formulations may be a practical option when injection-site pain affects treatment acceptability.

## Data Availability

The raw data supporting the conclusions of this article will be made available by the authors, without undue reservation.
